# Advancing the future of physical activity guidelines in Canada: an independent expert panel interpretation of the evidence

**DOI:** 10.1186/1479-5868-7-41

**Published:** 2010-05-11

**Authors:** Antero Kesäniemi, Chris J Riddoch, Bruce Reeder, Steven N Blair, Thorkild IA Sørensen

**Affiliations:** 1 Institute of Clinical Medicine, Department of Internal Medicine and Biocenter Oulu, University of Oulu, PO Box 5000, FIN-90014, Finland; 2 School for Health, University of Bath, 6.4 Norwood House, Bath BA2 7AY, UK; 3 School of Public Health, Department of Community Health and Epidemiology, University of Saskatchewan, 107 Wiggins Road, Saskatoon, Saskatchewan S7N 5E5, Canada; 4 Department of Exercise Science, Public Health Research Building, University of South Carolina, 225, 921 Assembly Street, Columbia, South Carolina, 29208, USA; 5 Institute of Preventive Medicine, Copenhagen University Hospital, Centre for Health and Society, Øster Søgade 18, 1st fl, DK-1357 Copenhagen K, Denmark

## Abstract

The Canadian Society for Exercise Physiology, in partnership with the Public Health Agency of Canada, has initiated a review of their physical activity guidelines to promote healthy active living for Canadian children, youth, adults and older adults; previous guidelines were released in 2002, 2002, 1998 and 1999 respectively. Several background papers from this project were published recently and provide foundation evidence upon which to base new guidelines. Furthermore, comprehensive systematic reviews were completed to ensure a rigorous evaluation of evidence informing the revision of physical activity guidelines for asymptomatic populations. The overall guideline development process is being guided and assessed by the AGREE II instrument. A meeting of experts was convened to present the evidence complied to inform the guideline revisions. An independent expert panel was assembled to review the background materials and systematic reviews; listen to the presentations and discussions at the expert meeting; ask for clarification; and produce the present paper representing their interpretation of the evidence including grading of the evidence and their identification of needs for future research. The paper includes also their recommendations for evidence-informed physical activity guidelines.

## Review

There is extensive research supporting the importance of regular physical activity (PA) in reducing all-cause mortality and improving several health outcomes. Canada has played an important role in the development of PA guidelines and guides for individuals of all ages. In 1998, the Canadian Society for Exercise Physiology (CSEP), in partnership with Health Canada (now the Public Health Agency of Canada) and others, released *Canada's Physical Activity Guide to Healthy Active Living *for adults between the ages 20 and 55 years [1]. This was followed by *Canada's Physical Activity Guide to Healthy Active Living for Older Adults *in 1999 and *Canada's Physical Activity Guides for Children and Youth *in 2002 [2,3]. Research in the PA sciences has advanced remarkably since these publications. Therefore, there has been a need to review the current research evidence to determine whether updated guidelines are required to help Canadians improve their health through regular PA.

In 2007 CSEP, with support from the Public Health Agency of Canada, completed an extensive review of PA measurement and guidelines in Canada, with 14 background papers published as a combined supplement of the *Canadian Journal of Public Health *and *Applied Physiology, Nutrition, and Metabolism *[4]. Further to this, on January 14-16, 2009, CSEP and the Public Health Agency of Canada organized an international consensus conference to discuss the current research evidence in asymptomatic individuals (i.e., with no pre-existing conditions health conditions) and to determine possible modifications to the existing guidelines. At the conference, experts presented evidence from detailed systematic reviews in which they updated findings on PA and health outcomes, including dose-response relationships and possible adverse effects of PA [5-9]. Detailed evidence of the health benefits of PA in school-aged children and youth [5], adults [6], older adults [7], Aboriginal populations, individuals with a disability, pre-school children, and pregnant women were presented. A rigorous, evidence-based approach was used to develop the levels of evidence for the relationship between PA and health outcomes using the procedures adopted by the Canadian Obesity Guidelines [10].

An independent expert panel (the Consensus Panel) comprised of five individuals (two with subject-specific knowledge in PA and health (CR, SB) and three with expertise from the associated disciplines of public health (BR), general and clinical epidemiology (TS), and medicine (AK)) was tasked with interpreting and rating the evidence compiled and presented for the first three categories mentioned (school-aged children and youth, adults, and older adults). The Consensus Panel reviewed and evaluated the evidence presented, assigned it to an evidence category (according to the classification systems shown in Tables 1 and 2), and identified needs for future research. For the determination of the dose-response relationship between PA and health outcomes, the authors identified studies that evaluated the relationship between at least three different levels of PA. The Consensus Panel agreed with this approach for the determination of the shape of the curve between PA and health outcomes, but also considers the information based on only two levels of PA informative.

**Table 1:** Criteria for assigning a level of evidence to recommendations

Level of Evidence	Criteria
Level 1	Randomized control trials without important limitations
Level 2	Randomized control trials with important limitations
	Observational studies (non-randomized clinical trials or cohort studies) with overwhelming evidence
Level 3	Other observational studies (prospective cohort studies, case-control studies, case series)
Level 4	Inadequate or no data in population of interest
	Anecdotal evidence or clinical experience

**Table 2:** Criteria for assigning a grade to recommendations

Grade of Evidence	Criteria
Grade A	Strong recommendation (action can apply to most individuals in most circumstances) Benefits clearly outweigh risks (or vice-versa) Evidence is at Level 1, 2, or 3
Grade B	Weak recommendation (action may differ depending on individual's characteristics or other circumstances)
	Unclear if benefits outweigh risks Evidence is at Level 1, 2, or 3
Grade C	Consensus recommendations (alternative actions may be equally reasonable)
	Unclear if benefits outweigh risks Evidence is at Level 3 or 4

In preparing the current document, the Consensus Panel observed that some important information was not available in the systematic reviews presented. Therefore, the Consensus Panel has used additional sources of information to cover these areas. Accordingly, the Consensus Panel's consensus statement, presented here, is based on (1) the background articles published in the 2007 supplement [4], (2) the systematic reviews presented at the consensus conference [5-9], and (3) additional information available in the literature (4) discussion and debate at the international consensus conference. This consensus statement provides information and recommendations for school-aged children and youth, adults, and older adults, and a discussion of the adverse effects of PA and needs for future research. Short summaries of the evidence are provided for each set of recommendations, with complete details available in the source documents [4-9]; the purpose of this paper is to provide a short summary, independent interpretation and list of recommendations based on the detailed work previously done. All of the recommendations presented herein represent the unanimous views of the panel. Because of the heterogeneity of data across studies, we did not directly link the evidence to individual studies, and refer the reader to each of the systematic reviews. Details on the history of this project and an assessment of the quality and rigor of processes informing these recommendations are provided in the paper by Tremblay et al [11].

## School-aged children and youth, aged 5-19 years

### Introduction

The systematic review [5] examined evidence with respect to seven key health indicators known to be associated with either PA or fitness: blood cholesterol, blood pressure, overweight/obesity, bone mineral density, metabolic syndrome, depression, and injuries. After removing duplicates, a total of 437 articles were identified for cholesterol, 1,151 for depression, 2,505 for injuries, 1,181 for bone mineral density, 1,677 for blood pressure, 5,824 for obesity, and 1,677 for the metabolic syndrome, for a total of 14,452. Many of these articles were retrieved for two or more health outcomes, and after removing these duplicates there was a total of 11,088 unique citations. After review of the titles and abstracts, full-text copies of 454 citations were obtained and reviewed. Of these, 86 passed the eligibility criteria, reported dichotomous outcomes, and were included in the systematic review. Several of the papers included had results for two or more of the health outcomes.

Evidence was reviewed with respect to the following questions:

1. How much (volume) PA is needed for minimal and optimal health benefits?

2. What types of PA are needed to produce health benefits?

3. What is the appropriate PA intensity?

4. Does the effect of PA on health in school-age children and youth vary by sex and/or age?

The current recommendations within Canada's PA Guide for Children and Youth are as follows [3]:

1. Increase the time currently spent on PA, starting with 30 minutes/day more, and progress over approximately 5 months to 90 minutes/day more.

2. Build up PA throughout the day in periods of at least 5-10 minutes.

3. The 90-minute increase in PA should include at least 1 hour of moderate activity (e.g., brisk walking, skating, bicycle riding) and 30 minutes of vigorous activity (e.g., running, basketball, soccer).

4. Combine three types of physical activities--endurance, flexibility, and strength activities--to achieve the best results.

5. Reduce non-active time spent watching television and video, playing computer games, and surfing the internet. Start with 30 minutes/day less of such activities and progress over the course of approximately 5 months to 90 minutes/day less.

A brief summary of the current evidence of the associations between PA/fitness and health outcomes for children and youth is presented below.

### Associations with health outcomes

#### Cholesterol and blood lipids

A total of nine articles examining blood lipids and lipoproteins met the inclusion criteria, one of which was observational in nature and eight of which were experimental. Interventions ranged from 6 to 24 weeks in duration and included from 1 to 4 hours/week of prescribed exercise (mean: 9-34 minutes/day). Most of the exercise programs were aerobic in nature and included various forms of moderate- to vigorous-intensity PA.

The one observational (cross-sectional) study indicated that unfit girls (bottom quintile of fitness) were 1.89 times (95% confidence interval [CI] 1.12-3.17) more likely to have hypercholesterolemia than moderately and highly fit girls. Unfit boys were 3.68 times (95% CI 2.55-5.31) more likely to have hypercholesterolemia than moderately and highly fit boys.

Three of the eight experimental studies reported that exercise training had significant beneficial effects on at least one lipid/lipoprotein variable. These studies tended to prescribe aerobic exercise. Other studies, which included interventions based on resistance training and circuit training, reported small and/or insignificant findings for the variables examined. The interventions were predominantly found to be favorable in "high-risk" participants (e.g. those with obesity, high cholesterol).

The nature of the dose-response relationship between PA and blood lipids remains unclear. The effects of age and sex have not been adequately studied.

#### Blood pressure

Three observational studies (two cross-sectional studies and one prospective cohort study) and eight experimental studies were included. Interventions ranged from 4 to 25 weeks in duration. The experimental studies were limited to children and youth with high blood pressure or obesity. With one exception, interventions included between 60 and 180 minutes/week of prescribed exercise (mean: 9-30 minutes/day). Most of the exercise programs were aerobic in nature and included various forms of moderate- to vigorous-intensity PA.

Within the observational studies, the relationship between PA or fitness and hypertension was weak, and in one case was not significant. Results from the intervention studies were positive, with the majority reporting modest but significant reductions (approximately 5-7%) in systolic blood pressure in response to exercise training. A few of the studies also reported modest but significant changes (approximately 4-6%) in diastolic blood pressure in response to exercise. Unlike the aerobic-based exercise programs, the two studies that employed resistance training did not report significant reductions in blood pressure.

Dose-response associations and the effects of age and sex have not been adequately studied.

#### Metabolic syndrome

The existence of true "metabolic syndrome" in children is debated. Nevertheless, elements of adult metabolic syndrome are known to cluster in children, as they do in adults. In children, a variety of definitions are used to describe the clustering of risk factors that comprise the metabolic syndrome in adults. In this review, obesity and cholesterol levels--two constituent components of the metabolic syndrome--are discussed as separate health outcomes. This section includes studies that have addressed clustering of any constituent components and those measuring the effect of PA on insulin levels and/or insulin resistance--a main element of the metabolic syndrome.

Nine observational studies (eight cross-sectional, one prospective) were included, including some with large and diverse samples of participants. There were nine experimental studies (six randomized controlled trials, three quasi-experimental), all but one of which was conducted in overweight/obese children. Interventions ranged from 6 to 40 weeks in duration and included anywhere from 80 to 200 minutes/week of prescribed exercise. Most of the exercise programs were aerobic in nature and included various forms of moderate- to vigorous-intensity PA.

In three cross-sectional studies using self-reported PA, associations between PA and the metabolic syndrome were either weak or modest in strength, and all were non-significant. By comparison, the two studies that used accelerometers to measure PA and the four studies that used direct measures of cardiorespiratory fitness all reported strong and significant associations with the metabolic syndrome. In the experimental studies, half of the studies reported significant improvements in metabolic syndrome indicators with increased PA. Both aerobic and resistance training programs had positive effects. Programs with longer durations (>20 weeks) tended to report stronger effects.

In the observational studies that used more precise measures of PA and/or fitness, clear dose-response relationships with metabolic syndrome indicators were observed. However, the shape of the curve (e.g., linear or curvilinear) was unclear. Comparison of risk estimates in boys and girls suggests that PA/fitness may be more preventive of the metabolic syndrome in boys.

The effects of PA dose, intensity, age and sex have not been adequately studied.

#### Overweight and Obesity

Thirty-one observational studies (25 cross-sectional, three prospective cohort, two case-control, one mixed) and 25 intervention studies (13 randomized controlled trials, 12 quasi-experimental) were included in this review. The studies ranged in length from 1 month to 2 years, with most being 4-6 months in duration. The amounts of exercise prescribed typically ranged from 2 to 3.5 hours/week (mean: 17-30 minutes/day). Approximately half of the studies were limited to overweight/obese participants. Observational studies using self-report measures of PA tended to report weak to moderate inverse associations of PA with overweight/obesity, with many risk estimates being non-significant. Eight studies using either objective measures of PA (one pedometer, three accelerometer) or cardiorespiratory fitness reported significant associations that were modest to strong in magnitude. Studies that were limited to more vigorous forms of exercise tended to produce stronger effect estimates. The majority of studies involving exercise interventions that were aerobic in nature observed significant changes in measures of total fat and abdominal fat in response to training. Changes in body-mass index and weight were far less consistent in these studies. Most studies that employed other training modalities (four resistance training, two circuit training, one Pilates) did not observe significant improvements in measures of total or abdominal fat in response to training.

There is good evidence of an inverse dose-response association between PA and being overweight or obese, but the shape of the curve is unclear because both linear and curvilinear patterns were observed. Visual inspection of risk estimates suggests that PA and fitness are more strongly associated with weight in boys than in girls.

#### Bone mineral density

No observational studies in the literature search met the systematic review criteria of presenting the effects of PA on bone mineral density as a dichotomous outcome. Eleven experimental studies were included. The programs typically consisted of moderate- to high-intensity anaerobic, high-impact activities such as jumping. These programs were performed from 3 to 60 minutes in length on at least 2 or 3 days of the week, and lasted from a few months to 2 years in duration.

As little as 10 minutes of moderate- to high-impact activities performed on as little as 2 or 3 days of the week was found to have a positive impact on bone mineral density when combined with more general weight-bearing aerobic activities that are also beneficial for cardiovascular risk factors and obesity prevention (e.g., jogging, play).

Dose-response relationships and age and gender differences have not been adequately studied.

#### Depression/depressive symptoms

Three observational (all cross-sectional) and three experimental studies (two randomized controlled trials) were included. All of the experimental studies prescribed aerobic exercise. Programs for children with depressive symptoms were approximately 8-12 weeks in length and prescribed modest amounts of exercise (60-90 minutes/week).

The observational studies reported small and insignificant or modest relationships between PA and depression. Two of the experimental studies observed significant improvements in depressive symptoms. One of the studies included both high-intensity and moderate-intensity PA programs, and only the high-intensity program resulted in significant improvements in depression scores in comparison to the control group, which performed flexibility exercises.

Dose-response associations and age and gender differences have not been adequately studied.

#### Injuries

Three cross-sectional studies were included. These studies focused on participants who had been injured or groups composed entirely of athletes (e.g., football players, ballet dancers), and addressed medically treated injuries, the severity of which was poorly reported.

All three of the studies reported higher rates of injury in physically activity children and youth. There was clear evidence of a dose-response relationship between PA participation and the likelihood of injury. That is, as the PA level increased, the likelihood of injury increased in a graded fashion. More vigorous activity seemed to be related to high rates of injury.

Age and gender differences have not been adequately studied.

### Recommendations

Based on the 2007 supplement [4], the evidence presented at the conference, the systematic review [5] and other materials, the Consensus Panel makes three recommendations for school-age children and youth. Following these recommendations should stimulate sound growth and development and confer protection against known risk factors for adult chronic disease.

#### Recommendation 1

Children and youth aged 5-19 years of age should accumulate at least 1 hour and up to several hours of at least moderate-intensity PA on a daily basis to achieve most of the health benefits associated with PA (evidence: level 3, grade A). Some health benefits can be achieved through 30 minutes/day of moderate-intensity PA, and this should be used as a "stepping stone" for currently sedentary children (evidence: level 2, grade A).

##### Interpretation of evidence and justification

With the exception of injuries, the collective evidence from observational studies for several health outcomes suggests that the majority of health benefits are achieved at the higher end of the PA spectrum. Normal lifestyle-embedded PA needs to be added to the purposeful PA most often used for the intervention studies. This justifies the optimal level of PA as ≥ 1 hour/day, despite the lack of experimental evidence or randomized trials. Experimental studies for several health outcomes suggest that participating in as little as 2 or 3 hours/week of at least moderate-intensity PA is associated with health benefits. This is supported by evidence from observational studies that have demonstrated dose-response relationships between PA and health outcomes, with differences in health risk between the least active (or fit) and the second least active groups. Thus, it would seem appropriate to set a minimal "stepping stone" level of 30 minutes/day of moderate-intensity activity, which will confer some health benefits.

#### Recommendation 2

Vigorous-intensity activities should be incorporated or added when possible, including activities that strengthen muscle and bone (evidence: level 3, grade B).

##### Interpretation of evidence and justification

Although few studies have systematically evaluated the differential effects of various intensities of PA, the available information suggests that vigorous-intensity activities may provide additional health benefits beyond moderate-intensity activities. Furthermore, many of the experimental studies that observed significant changes in health variables prescribed exercise that would fall within the vigorous-intensity range (≥ 7 METS) or in the upper end of the moderate-intensity range (5-7 METS).

#### Recommendation 3

Aerobic activities should make up the majority of the daily PA. Muscle- and bone-strengthening activities should be incorporated on at least 3 days of the week (evidence: level 2, grade A).

##### Interpretation of evidence and justification

Many of the health outcomes examined, particularly obesity and measures of cardiometabolic health, responded almost exclusively to aerobic-type activities. However, bone health was more favorably effected by modest amounts of resistance training and other high-impact activities (e.g., jumping) that were performed on at least 2-3 days of the week. It should be noted that the evidence supports the applicability of these recommendations through an age range of 5-19 years.

## Summary

There is a level of consistency for the beneficial effects of PA across health outcomes. Some more recent, large, epidemiological studies that have used more robust measurement methods have suggested the strongest associations. The main recommendations (recommendations 1 and 2) reflect the linear or curvilinear dose-response association observable for some of the main health outcomes. In general, the three recommendations are justified by the level and strength of the evidence. Although not reviewed here, it is likely that reducing sedentary behaviour is important for health. The Canadian Paediatric Society recommends a maximum of 2 hours/day of television-viewing time [12]. We endorse that recommendation.

## Adults aged 19-65 years

### Introduction

To assess the physical activity guidelines for asymptomatic adults a systematic, evidence-based approach was taken by the Consensus Panel. The evidence considered included the 2007 supplement [4], the systematic review presented at the consensus conference[6], and reviews conducted by scientists in the US [13] and UK [14]. The systematic review critically evaluated scientific literature published in English from January 1966 to March 2008 that examined the relationship between PA and all-cause mortality or PA and the incidence of one or more of seven chronic diseases (cardiovascular disease [excluding stroke], stroke, hypertension, colon cancer, breast cancer, type 2 diabetes, and osteoporosis) in adults aged 19-65 years. Any measure of PA (e.g., self-report, pedometer, accelerometer) or fitness (e.g., maximal aerobic power (VO2 max) was eligible for inclusion; however, only studies that presented at least three different levels of PA were included.

In adults, PA has been promoted for health benefits since the release of the *Canada's Physical Activity Guide to Healthy Active Living *in 1998 [1]. With the present review, the Consensus Panel examined evidence for the following:

1. The relationship between PA and all-cause mortality.

2. The relationship between PA and the incidence of seven chronic conditions: cardiovascular disease (excluding stroke), stroke, hypertension, colon cancer, breast cancer, type 2 diabetes, and osteoporosis. These conditions were identified *a priori *as diseases of major public health importance for which an evidence base exists.

3. The dose-response relationship between PA and these health outcomes.

This review focuses on the primary prevention of the seven conditions in asymptomatic adults, not on the role of PA in secondary prevention or treatment. Canada's current recommendations for PA for adults aged 20-55 years are as follows [1]:

1. Accumulate 20-60 minutes of PA every day in periods of at least 10 minutes each. The time needed depends on effort: light effort 60 minutes, moderate effort 30-60 minutes, and vigorous effort 20-30 minutes.

2. Perform three types of activity to keep your body healthy: endurance activities (4-7 days a week), flexibility activities (4-7 days a week), and strength activities (2-4 days a week).

A brief summary of the current evidence of the associations between PA/fitness and health outcomes for adults is presented below.

### Associations with health outcomes

A total of 261 articles met the eligibility criteria. These examined the relationships between PA/fitness and premature all-cause mortality (*N *= 72), cardiovascular disease (*N *= 51), stroke (*N *= 25), hypertension (*N *= 13), colon cancer (*N *= 34), breast cancer (*N *= 43), type 2 diabetes (*N *= 20), and osteoporosis (*N *= 2).

#### All-cause mortality

A total of 2,040 relevant citations were identified, with 72 articles eligible for inclusion. These studies followed over 1,600,000 participants for an average of 11.7 years, and identified over 111,000 deaths (all-cause). The vast majority of studies used a prospective cohort design.

**Figure 1:**
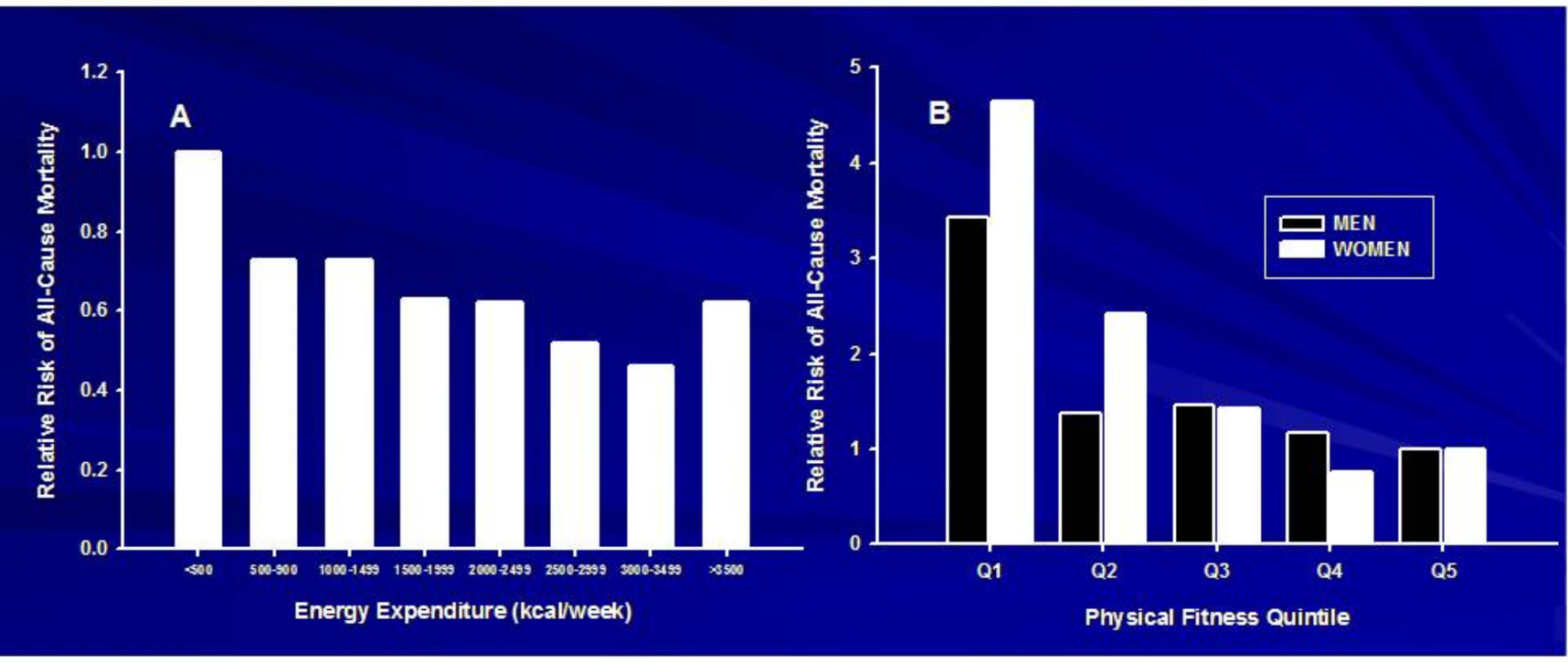
**Early investigations examining the relative risks of all-cause mortality**. Early investigations examining the relative risks of all-cause mortality as a function of (A) physical activity (data from Blair et al. [17] and (B) fitness level (data adapted from Paffenbarger et al. [16]).

A strong and consistent inverse relationship between PA and all-cause mortality was observed in both men and women, with a mean of 31% lower risk in the most active compared to the least active group. In studies that used objective measures of aerobic fitness, the risk reduction averaged 50%. The current Canadian guidelines (gross energy expenditure of approximately 4.2 MJ/week, 1,000 kcal/week) are estimated to be associated with at least a 20% lower risk for premature all-cause mortality [15-17]. An inverse curvilinear dose-response relationship is seen with both PA and fitness (Figure 1).

#### Cardiovascular disease

The associations of PA with stroke and other cardiovascular disease were examined separately. For cardiovascular disease, a total of 9,408 citations were identified and 51 articles deemed eligible for inclusion in the systematic review. These studies followed 658,747 participants for an average of 14.6 years and observed 30,292 incident cases of disease. A consistent inverse relationship between PA and cardiovascular disease was seen, with a mean of 33% lower risk in the most active compared to the least active groups. This risk reduction was observed in both men and women and in Caucasian as well as non-Caucasian populations. In studies that used objective measures of aerobic fitness, the risk reduction averaged 50%.

#### Stroke

A total of 1,104 citations that examined the relationship between PA and stroke were identified and 25 articles were deemed eligible for inclusion in the systematic review. These prospective cohort studies followed 479,336 participants for an average of 13.2 years and observed 12,361 cases of stroke. As with other cardiovascular disease, an inverse relationship was noted between PA and stroke, with a mean of 31% lower risk in the most active compared to the least active group. Greater risk reductions were observed in studies that measured fitness than in those that measured PA. The shape of the dose-response relationship between PA and stroke varied between studies, and it is uncertain if the inverse relationship seen with total and ischemic stroke also applies to hemorrhagic stroke.

#### Hypertension

A total of 6,287 citations were identified that examined the relationship between PA and hypertension and 13 studies were included in the systematic review. These primarily prospective cohort studies followed 113,524 participants for an average of 8.7 years and observed 11,695 cases of hypertension. An inverse relationship was seen between PA and the incidence of hypertension, with the most active individuals experiencing a mean of 32% lower risk than the least active. The shape of the dose-response relationship, however, was unclear. Some evidence from the review and randomized controlled trials suggests that moderate to vigorous activity is required to produce an observable reduction in the incidence of hypertension. Furthermore, a body of evidence indicates that moderate-intensity resistance training can reduce blood pressure.

#### Colon cancer

A total of 252 citations were identified that examined the relationship between PA and the incidence of colon cancer and 34 articles were included in the systematic review. These case-control, prospective cohort, and cross-sectional studies used diverse methods to measure both occupational and leisure-time PA. The studies involved a total of 1,322,900 participants and 14,625 cases of colon cancer. The length of follow-up in the 11 prospective cohort studies averaged 10.7 years. Although considerable variability in study findings is seen, those in the most active groups had, on average, a 30% lower risk of developing colon cancer than the least active. The shape of the dose-response relationship is unclear.

#### Breast cancer

A total of 571 citations were identified that examined the relationship between PA and the incidence of breast cancer and 43 articles were included in the systematic review. These prospective cohort and case-control studies followed 1,860,660 participants and observed 80,138 cases of breast cancer. Follow-up for the prospective cohort studies averaged 10.5 years. An inverse relationship was seen between PA and the incidence of breast cancer, with the most active individuals experiencing, on average, a 20% lower risk than the least active. The majority of evidence suggests that 30-60 minutes/day of moderate- to vigorous-intensity activity is required to reduce the incidence of breast cancer, and that the relationship is stronger in post-than in pre-menopausal women.

#### Type 2 diabetes

A total of 3,655 citations were identified that examined the relationship between PA and the incidence of type 2 diabetes and 20 articles were included in the systematic review. These prospective cohort studies followed 624,952 participants for an average of 9.3 years and observed 19,325 cases of diabetes. A consistent inverse relationship was observed between levels of PA or fitness and type 2 diabetes. When comparing the most active/fit to the least active/fit group, an average of 42% risk reduction is seen. An inverse curvilinear dose-response relationship exists. Although it is difficult to separate exercise volume from intensity in the evidence available, small changes in PA levels yield substantial reductions in the risk of developing diabetes. Randomized controlled trials of individuals at high risk for the development of diabetes suggest that 150 minutes/week of moderately vigorous activity results in a 58% reduction in the risk of developing diabetes.

#### Osteoporosis/bone mineral density

Numerous exercise intervention trials and systematic reviews have demonstrated that aerobic and resistance activities enhance bone mineral density across the lifespan and reduce the incidence of fractures. However, little research has examined the relationship between PA and the incidence of osteoporosis. Although 3,655 studies were identified in the systematic review only 2 were included. These studies revealed an inverse relationship between PA level and the prevalence of osteoporosis.

### Recommendations

Based on the 2007 supplement [4], the evidence presented at the conference, the systematic review [6] and other materials [13,14], the Consensus Panel makes three recommendations for adults aged 19-65 years.

#### Recommendation 1

Adults aged 19-65 years should accumulate 150 minutes/week of moderate-intensity PA or 90 minutes/week of vigorous-intensity PA in periods of at least 10 minutes each. Greater amounts of activity and more vigorous activity provide additional benefits (evidence: level 2, grade A).

##### Interpretation of evidence and justification

The evidence indicates that 150-180 minutes/week of moderate or 90 minutes/week of vigorous PA is associated with a 30% reduction in the risk of all-cause mortality and reductions in the incidence of cardiovascular disease, stroke, hypertension, colon and breast cancer, and type 2 diabetes. The inverse dose-response relationship seen with these conditions demonstrates that additional health benefits are observed with greater amounts of or more vigorous PA. The greatest benefit is seen when the PA is distributed throughout the week and in periods of at least 10 minutes' duration. This volume of exercise may also prevent weight gain in some individuals; however, greater volumes may be required for others.

#### Recommendation 2

Engage in resistance activities on 2-4 days/week (evidence: level 2, grade A).

##### Interpretation of evidence and justification

Recent reviews [4,13-15] have demonstrated that musculoskeletal fitness improves blood pressure, bone mineral density, mobility and functional independence, and overall quality of life, and reduces premature mortality and the risk of falls.

#### Recommendation 3

Engage in flexibility activities on 4-7 days/week (evidence: level 3, grade A).

##### Interpretation of evidence and justification

The inclusion of flexibility activities as part of an adult's PA routine may enhance mobility and functional independence, and decrease the risk of falls.

## Older adults aged ≥ 65 years

### Introduction

The Consensus Panel reviewed several sources of evidence regarding PA and health in older adults (≥ 65 years). These included the 2007 supplement [4,18], systematic reviews presented at the consensus conference [7], and reviews conducted by scientists in the US [13] and UK [14]. Collectively, these sources provide an extensive and thorough review of the literature on PA and health.

Canada's current recommendations for PA for adults aged ≥ 64 years are as follows [2]:

1. Increase endurance activities--4-7 days a week.

2. Increase flexibility activities--daily.

3. Increase strength and balance activities--2-4 days a week.

A brief summary of the current evidence of the associations between PA/fitness and health outcomes for older adults is presented below.

### Associations with health outcomes

#### Morbidity from chronic disease and all-cause mortality

**Figure 2:**
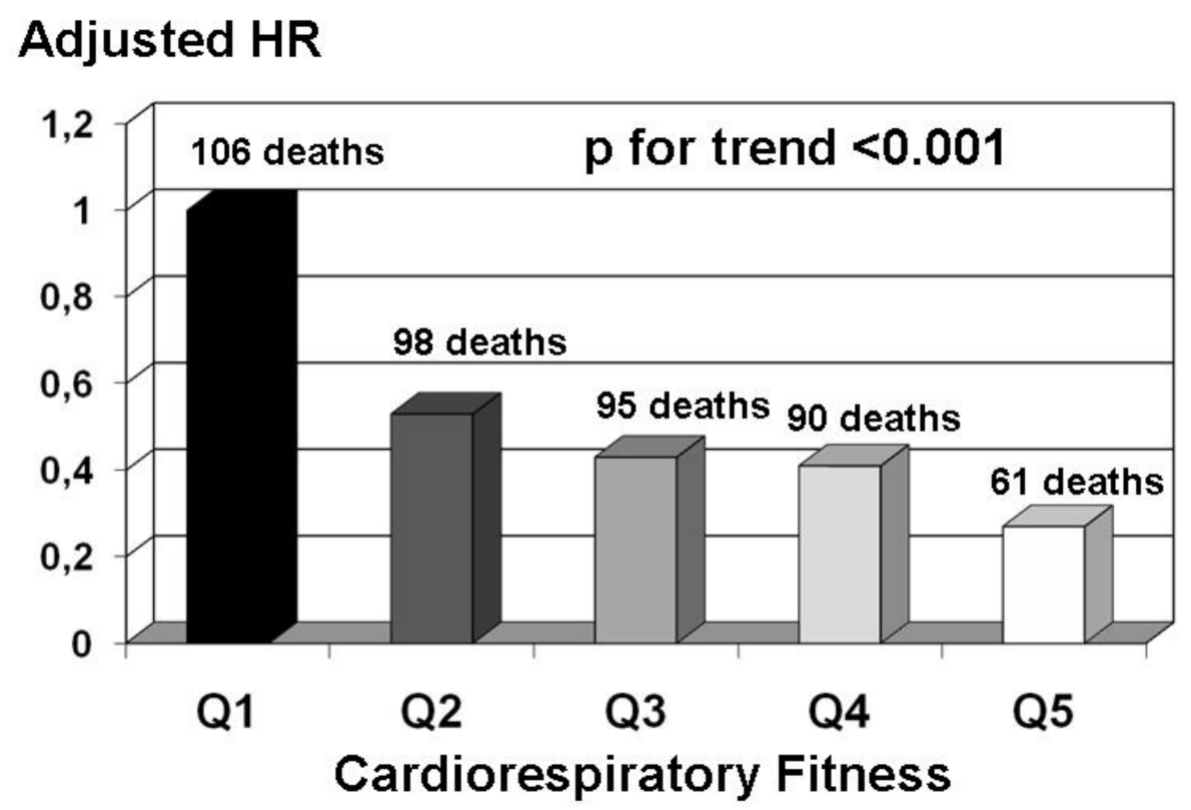
**Adjusted hazard ratios for all-cause mortality by fitness group**. Multivariate (adjusted for age, examination year, smoking, abnormal exercise electrocardiogram, baseline health conditions) and percentage body fat-adjusted hazard ratios (HRs) for all-cause mortality by fitness group, Aerobics Center Longitudinal Study in 2,603 adults aged ≥ 60 years (Adapted from data by Sui et al. [19]).

Extensive reviews of studies on PA/fitness and various health outcomes have been published [4,13-15]. These reviews reported strong, consistent, and biologically plausible evidence for the benefits of regular PA on numerous health outcomes. There is a dose-response gradient for morbidity and mortality across levels of PA. An example for cardiorespiratory fitness and all-cause mortality is shown in Figure 2[19]. With the least fit quintile as the reference group, individuals in the second quintile of fitness had approximately a 50% lower mortality risk. The risk of death continued to decline across other fitness groups, with the most fit 20% having approximately a 79% lower risk compared with the referent group. Numerous other studies [6,7,13-18] have shown similar dose-response relationships between PA or fitness for a variety of health outcomes, although the gradient tends to be steeper in fitness studies than in PA studies (see Figure 1). This is perhaps due to greater misclassification of the exposure to PA in the latter studies.

#### Functional independence and disability

Low levels of PA in older adults are likely to lead to a decline in cardiorespiratory fitness and muscular strength that may reach levels that are incompatible with some aspects of functional independence. Thus, it is important to maintain cardiorespiratory fitness and muscular strength to prevent crossing "functional thresholds" [7].

A systematic review that evaluated the relationship of the PA of healthy, community-dwelling older (>65 years) adults with outcomes related to functional limitations, disability, or loss of independence was presented at the consensus conference [7]. The review included epidemiological investigations and exercise-training intervention studies. A total of 66 articles on the relationship between PA and functional independence met the inclusion criteria for the review, and 34 articles included cognitive function as an outcome. The review did not include persons who, at baseline, had clinical conditions, functional limitations, disability, or frailty or who were >85 years of age. PA was assessed by self-report in most studies. Most studies presented data in two PA groups, but a few included three or four levels of PA. Two studies included cardiorespiratory fitness as the exposure. A few studies assessed muscular strength. Functional outcomes included self-reported functional ability or disability, self-reported PA limitations, and performance measures. Outcomes were typically reported in dichotomous categories. The review also included 35 prospective studies, with sample sizes ranging from 200-10,000 individuals. Follow-up ranged from 2.5 to 30 years. Physically active individuals had an approximately 50% lower risk of disability in activities of daily living and instrumental activities of daily living, with only two negative studies. Studies with more than two categories of PA tended to show an inverse dose-response trend.

Physically active individuals also tended to have a slower rate of functional decline, with those who were active at approximately 50% lower risk compared to the inactive. There was consistency across studies and across a wide range of outcome measures. The benefits were observed in both short-term and long-term follow-up. There was some evidence of a dose-response relationship.

Fourteen exercise-training studies were reviewed, and they were consistent in showing improved functional performance in the exercise groups. A majority of the studies used moderate-intensity aerobic PA as the intervention, typically walking on at least 3 days/week. Some studies also combined aerobic exercise with resistance training, and reported greater functional improvements with combined aerobic and resistance programs versus a resistance or aerobic programs alone.

#### Cognitive function

The search found a total of 861 citations of which 32 met the inclusion criteria; an additional 2 papers were found from checking reference lists and the authors files, therefore 34 studies were included in the systematic review [7]. Cognitive measures varied widely across studies, and included reaction time, motor function, memory, executive function, and visual attention. Seventy-one percent (24/34) of the studies showed a positive relationship between PA and some aspect of cognitive function. Prospective studies indicated a reduced risk of dementia and Alzheimer's disease in physically active individuals. The majority (7/12) of the exercise-training studies showed a positive effect on at least one cognitive outcome.

### Recommendations

Based on evidence from the sources described the Consensus Panel makes three recommendations for PA for older adults. These recommendations apply to apparently healthy individuals and not to populations with clinical disease. Following the recommendations should reduce the risk of chronic disease, premature mortality, loss of function and disability, and cognitive decline.

#### Recommendation 1

Older adults should participate in moderate-intensity aerobic activity for a total of 150 minutes/week, or in vigorous-intensity activity for a total of 90 minutes/week. Moderate- and vigorous-intensity activities are defined as approximately 50% and 60-70% of maximal aerobic capacity, respectively. For many older adults, walking at 3.0-3.5 mph is a good example of moderate-intensity activity. The dose of PA described here is *in addition *to the routine and light-intensity activities of daily living, and can be expected to reduce the risk of several chronic diseases and premature death by 20-30%. Regular PA is also important for maintaining a healthy body weight. The PA recommendation presented here will be sufficient for many people to prevent weight gain, while others may need to engage in more PA to achieve this benefit. Obviously, caloric intake also must be considered as a tool for managing body weight.

Higher doses of PA, and more vigorous-intensity activity provide additional health benefits. The weekly dose of PA may be accumulated in sessions of at least 10 minutes of moderate-intensity activity (evidence: level 2, grade A).

##### Interpretation of evidence and justification

A previous publication by Paterson et al. [18] and the systematic review presented at the consensus conference [7] provide strong evidence of the benefits of aerobic PA for older adults. The evidence comes from large cohort studies and controlled exercise trials. The evidence is highly consistent, with few negative studies. Regular aerobic PA has an inverse dose-response relationship with major chronic diseases (coronary heart disease, type 2 diabetes, depression, some cancers, dementia, disability, and loss of function). The recommended dose of aerobic PA reduces the risk of these conditions and functional limitations by 30-50%, and higher doses of PA provide further benefits.

#### Recommendation 2

In addition to the aerobic PA recommendation, older adults should also engage in resistance exercises on 2 days/week. Resistance exercise should involve the major muscle groups of the body, and should consist of 8-12 repetitions at >60% of 1 repetition maximum (RM). Daily activities that involve lifting, carrying, and pushing tasks should be maintained because they can also benefit muscular and bone health (evidence: level 2, grade A).

##### Interpretation of evidence and justification

A previous publications by Paterson et al. [18] and a systematic review presented at the consensus conference [7] provide evidence for the benefits of resistance exercise in older adults. The major benefit of resistance training is preservation of muscle mass and prevention of age-related sarcopenia. The evidence supports the potential benefits of resistance training in preventing chronic diseases such as the metabolic syndrome and in delaying mortality. Greater strength--and particularly muscle power--results in maintaining function and potentially preventing disability.

#### Recommendation 3

Good balance helps prevent falls, and older adults should participate in activities that improve and maintain balance. Such activities include dancing, walking on uneven surfaces such as a field or in a forest, and various exercises such as standing on one leg. Stretching exercises should be done regularly to maintain good flexibility. Inflexibility can interfere with routine daily tasks and in participation in leisuretime PA (evidence: level 2, grade A).

##### Interpretation of evidence and justification

There is growing evidence that balance activities result in a lower risk of falling, a major health concern for more frail older adults. Balance training, along with activities to strengthen the muscles of the legs, is the best strategy to reduce falls and complications from falls. Stretching exercises will help maintain joint flexibility, which can help maintain function.

## Adverse effects

There are extensive health benefits of regular PA, as discussed in detail in the three main sections of this paper. However it is important to be cognizant of the potential adverse events of PA. Fortunately, the risk of adverse events is low, especially for those doing moderate amounts and intensities of PA. The *2008 Physical Activity Guidelines for Americans *report that musculoskeletal injuries are the most common adverse event, but that the injury rate is approximately one event for every 1,000 hours of walking for exercise [13]. It is a bit higher, at four events per 1,000 hours, for running. Serious adverse events, such as cardiac arrest, are more likely during exercise than at rest, but are still relatively rare. Although serious adverse events are higher during the exercise period, the overall risk for regular exercisers is still substantially lower over the entire day than it is for non-exercisers. Thus, the benefits of regular PA outweigh the risks [13].

Individuals need to be aware of the potential risks of physical activity and should consult their primary-care provider if they have questions. Individuals should also be aware of the early warning signs of potential serious adverse events, such as chest pain or discomfort with exercise, dizziness, abdominal pain, or other major symptoms. In such cases, a medical care authority should be consulted. In addition, the risks of exercising with or shortly after a febrile infection should be understood and prevented.

## Future research

The preparatory work for the revision of the Canadian guidelines has led to the identification of numerous questions for which adequate answers are not available in the literature and that warrant further research. Many of the questions raised here are of potentially great interest to extending our understanding of the relationship between PA and health. This section has restricted proposals for future research to questions that were felt to provide results that will improve the basis for future revisions of the PA guidelines.

### General methodological issues

Because guidelines for PA aim at improving PA in segments of the population where the it is inadequate, there is clearly a need for more studies that provide solid evidence for how to make changes in the best way, and evidence for the health effects of these changes. Many of the studies that have formed the evidence for these guidelines did not directly address these fundamental questions and, moreover, were of rather poor or suboptimal quality for a variety of reasons. Thus, there is a need for more high-quality, large-scale, randomized intervention trials testing methods of increasing PA and testing the health effects of increasing PA where applicable.

For research questions that realistically cannot be addressed in randomized trials, such as the longterm effects of behavioural changes at the population level on morbidity and mortality, there is a similarly strong need for high-quality, large-scale, long-lasting, observational prospective cohort studies with repeated assessment of PA, allowing the identification of groups who have changed PA, confounders, and health outcomes. In both randomized trials and observational cohort studies, there is a need to apply far more precise and accurate measurement tools, especially for PA, but also for several health outcomes and potential confounders. Also, reporting of randomized clinical trials should be in accordance with the CONSORT guidelines or of non-randomized trials using the TREND guidelines [20].

### Population segments

Considering the heterogeneity of the population in Canada, investigations of corresponding differences between groups within the population are needed with regard to the distribution of PA, conditions for improving PA, and the health effects of given amounts and intensities PA, all of which may justify different guidelines for various population groups. Such groups could be defined, for example, by sex, age range, ethnicity, socioeconomic status, family setting, disability, puberty, or pregnancy. With advances in genomic research, there is a prospect of defining groups by genetic profile. Groups may also be defined by characteristics that themselves can be manipulated or modified, such as other lifestyle factors. The best possible epidemiological-statistical tools (e.g. interaction analysis) should be applied to investigate whether it is really necessary to make distinctions between groups.

It must be taken into account that the more groups into which the population is divided, the more difficult it is to validly demonstrate differential effects. It is a major challenge to identify the optimal cost-benefit balance between interventions customized to particular groups--or even tailored to particular individuals--versus fewer and more broadly applicable interventions. A need for different interventions may apply to individuals who already suffer from various diseases, but PA in such groups should be separated into PA aimed at general preventive effects among these individuals and PA aimed at specific therapeutic effects for these individuals as patients (clinical guidelines).

### Health outcomes

PA is related to numerous health outcomes, many of which constitute the core of the evidence for the PA guidelines. However, there are also many other outcomes of potential relevance for which the evidence is too weak to provide a compelling argument for recommendations. These include, for example, mental health and cognitive ability, especially in children, adolescents, and the elderly; common musculoskeletal disorders that also create great demands on the health care sector, such as osteoarthritis and osteoporosis with related fractures; and chronic medical disorders such as chronic heart failure and chronic obstructive pulmonary disease. Obviously, the guidelines that are applicable to a single individual should not differ for different health outcomes. The task is to integrate the evidence for different health outcomes and thereby allow recommendations that are generally applicable irrespective of differences in relationships to different health outcomes.

Research that translates PA-related health outcomes to quality-of-life measures is needed with regard to immediate, short-term and long-term effects. This applies to all age groups and draws attention to definitions of good and poor health, which may need to be specific for different age groups, especially for young children and the oldest adults. The consistent finding of an inverse association between allcause mortality and PA should be extended to quality-adjusted life years or disability-free life years.

### PA assessment

Characterization of PA requires measurements of frequency, duration, intensity, volume (intensity × duration), and mode, all that vary over time intervals from seconds to years. Most studies so far have been based on self-reported (for children, parent-reported) PA, often very crudely categorized data, and this information about PA obviously suffers from several types of severe measurement error. This leads to misclassifications of individuals' PA and hence to biases in the assessment of the relationship between PA and health outcomes, between putative determinants of PA and PA, and between interventions aimed at changing PA and achieved changes in PA. It is a great challenge to obtain evidence based on better measurement technology. The current options are various types of devices that continuously monitor body movements (e.g., accelerometers); however, although these do provide a more objective measure of PA, they still do not cover all aspects of PA. Cardiorespiratory fitness has been used as a proxy for the cumulative effects of PA in the preceding period, but is also dependent on other factors (e.g., genetic constitution). Furthermore, there is a need to study the impact of a sedentary lifestyle as a separate entity from typically assessed PA, based on the suspicion that there are both determinants of and effects of a sedentary lifestyle that are not simply equivalent to "low PA" [21,22]. NEAT (non-exercise activity thermogenesis) may play an important role in mediating, moderating or determining the relationship between PA or sedentary behaviour and health outcomes and deserves further investigation [23].

### Effect measurement and dose-response

The magnitude of effects and the dose-response relationship, both for given levels of PA and for changes in PA, needs more investigation based on better measurement technology. When assessing effects in general and their dependence on the starting level of risk, it is of particular importance to pay attention to the measures of effects (relative versus absolute effect, where the former is heavily dependent on the reference level of risk that defines the denominator of the measure). Assessment of dose-response functions requires specification of the definition of "dose" (better characterization of PA) and of "response," including the time relationship (e.g., immediate, delayed, or cumulative investigations of the dose-response relationships should also address various compositions of the "dose," for example whether very-low-intensity PA for longer periods can a substitute high-intensity PA for short periods. Statistically convincing distinctions between different shapes of dose-response curves, including determination of threshold effects, will require very large sample sizes.

### Mediators

In the context of analytical epidemiological research (in both observational and interventional studies) addressing cause-effect relationships, a mediator is defined as a modifiable factor or variable that is influenced by the cause in question and thereby induces the effect that through the mediator is attributable to the cause. Obviously, causes may induce effects both through and beyond mediation of a particular mediator. From a biological or psychological point of view, the mediation process can be considered as the "mechanism" or "pathway" by which the PA is linked upstream to determinants of PA and downstream to health effects. Understanding the mediation process may enhance the opportunities for interventions to improve PA, and for increased PA to improve health, not least because different types of PA may be related to different mediators [24,25]. The current evidence on mediation is rather limited, and there is a great need for more research, both regarding the mediation of interventions aimed at changing PA (particularly psycho-behavioural factors, e.g., self-regulation and self-efficacy) and the mediation of changes in PA on health outcomes (e.g., via biological coronary heart disease risk factors, cardiorespiratory fitness, energy expenditure). Demonstration of the associations between a putative mediator and the cause, and between this mediator and the effect, may not suffice as evidence for mediation.

### Hazards and adverse effects

Although the evidence so far indicates that there are few hazards or adverse effects of increasing PA or keeping a high level of PA, the possibility of adverse effects should be kept in mind in conducting future studies; for the sake of transparency, developing clinical practice guidelines and when building evidence for increasing PA.

### Special biases

In addition to the usual type of biases that affect randomized trials and cohort studies, some special biases need consideration--namely, those based on continuously operating behaviours, such as PA, as determinants of concurrent or later chronic disease health outcomes, such as obesity and coronary heart disease, respectively. Reverse causation implies that the disease process is already ongoing and, although not yet clinically diagnosed, may lead to changes in PA or associated confounders. For a condition such as obesity, there is the additional problem that this condition tends to fluctuate (statistically with the effect of regression toward the mean) and behavioural lifestyle factors (e.g., PA) may change in response to this fluctuation [26,27]. Therefore, an apparent association may be created between these lifestyle factors and subsequent changes in the opposite direction.

### Systematic review process

The systematic review process is of fundamental importance to the synthesis of evidence forming the basis for guidelines [11,28]. Where the outcome of a review can be supported by a meta-analysis providing pertinent quantitative effect estimates, this should be done. Adherence to internationally well-established standards for systematic reviews and meta-analyses, for example as developed and implemented by the Cochrane Collaboration, will increase the value and trustworthiness of the output of the process. Particular attention needs to be paid to the correspondence between the aim of the review and the criteria for literature selection in order to avoid missing potentially informative, high-quality studies. Where available, previously performed systematic and high quality reviews should be taken as the starting point so that the review process can take advantage of what has already been done by others, while of course maintaining a critical analytical approach [29].

### Scoring systems

The application and interpretation of scoring systems for the quality and message of the evidence to the output should be based on scales that are adapted to the type of evidence obtainable. The current scoring systems are typically based on an assessment of evidence that can be generated in large-scale therapeutic randomized trials (e.g., drug trials). However, it is not possible to use such trial designs when addressing questions about the long-term effects on mortality of behavioural preventive measures such as PA. Future research should explore how existing medical-oriented evidence assessment procedures require modification to accommodate population and lifestyle behavioural modifications. One example of such modifications is that assessment of the intervention by comparative trial design may have to be based on randomisation of a few clusters in which the intervention is set up by facilitating or encouraging physical activity in various ways in institutions or regions. Another possibly necessary modification is that the evaluation of the outcome rather than being based on changes in risk of clinically relevant endpoints, namely morbidity and mortality, may have to be based on what else would be considered as surrogate endpoints such as well being, improved fitness and strength, improved risk factor profile, e.g. improved blood pressure, blood lipids, glucose tolerance.

### Harmonization of physical activity guidelines

Guidelines for PA are being prepared by governmental and non-governmental organizations in many countries (e.g., US, UK) and by several international institutions (e.g., the World Health Organization). Future revisions of guidelines in all countries should take advantage--alongside the systematic reviews being conducted conditional on previous reviews--of comparisons between different guidelines in order to identify the reasons for differences (e.g., evidence, cultural, autonomy) and use this information to contribute to the improvement of future guidelines. In the process of such harmonization it would also be of great value to make the rationale and criteria applied for choosing particular thresholds for duration and intensity of recommended PA of particular types explicit, given that the available evidence seldom leads to unambiguous thresholds and that feasibility in the target populations necessarily must be considered as well.

### Healthy lifestyle approach

Lifestyle risk factors, such as PA, diet, smoking, alcohol intake and sleep, are clustered and possibly interact, both in terms of their determinants (lifestyle habits may substitute for each other) and effects on health. There is an obvious need for a healthy lifestyle approach in researching these factors and thereby forming the basis for more comprehensive guidelines that may be adequate for large segments of the population [30,31].

## Conclusions

Public health challenges exacerbated by low levels of physical activity and high levels of sedentary behaviour make the careful preparation and dissemination of physical activity guidelines particularly important. Research evidence completed since the release of Canada's Physical Activity Guidelines [1-3] suggests the guidelines were appropriate but may now need some minor revisions to remain evidence-informed. Almost all people without existing disease can benefit from an increase in physical activity, including predominantly aerobic activity, but also activities to improve strength/resistance/power and flexibility. Adverse effects of an increase in physical activity are relatively rare and usually minor, leading to a net health promoting effect. Further research, with better measurement methodology and improved study quality, is required to better describe and detail the relationships among PA, sedentary behaviour and health outcomes to continue to inform physical activity guidelines for populations and various population sub-groups.

## Competing interests

TIAS is collaborating with various industries on obesity research, see here http://www.ipm.regionh.dk/person/tias/Disclosures.html. The other authors declare that they have no competing interests.

## Authors' contributions

All authors had an equal role in the formulation and drafting of this manuscript. All authors have read and approved the final manuscript.
